# Sulfated *Undaria pinnatifida* Polysaccharide Promotes Endocytosis of Nano-Calcium Oxalate Dihydrate by Repairing Subcellular Organelles in HK-2 Cells

**DOI:** 10.3390/antiox12051015

**Published:** 2023-04-28

**Authors:** Xue-Wu Chen, Yu-Yun Zheng, Jian-Ming Ouyang

**Affiliations:** Institute of Biomineralization and Lithiasis Research, College of Chemistry and Materials Science, Jinan University, Guangzhou 510632, China

**Keywords:** *Undaria pinnatifida* polysaccharide, acidic groups, cell repair, crystal endocytosis, primary hyperoxaluria

## Abstract

The clinical manifestation of primary hyperoxaluria includes hyperoxaluria and recurrent urinary calculi. In this study, an oxidative damage model was constructed based on oxalate damage to the human renal proximal tubular epithelial cells (HK-2), and a comparative study was carried out on four different sulfated levels of *Undaria pinnatifida* polysaccharides (UPP0, UPP1, UPP2, and UPP3 with sulfate group [–OSO_3_^−^] contents of 1.59%, 6.03%, 20.83%, and 36.39%, respectively) on the repair of oxidatively damaged HK-2 cells. The results showed that after repair by UPPs, cell viability was enhanced, healing ability was improved, the intracellular superoxide dismutase level and mitochondrial membrane potential were increased, malondialdehyde, reactive oxygen species, and intracellular Ca^2+^ levels were reduced, cellular autophagy was reduced; lysosomal integrity was improved, and cytoskeleton and cell morphology were restored. The ability of repaired cells to endocytose nano-calcium oxalate dihydrate crystals (nano−COD) was enhanced. The activity of UPPs was closely related to their –OSO_3_^−^ content. A too high or too low –OSO_3_^−^ content was not conducive to polysaccharide activity, and only UPP2 exhibited the best cell repair ability and strongest ability to promote the cell endocytosis of crystals. UPP2 may be used as a potential agent to inhibit CaOx crystal deposition caused by high oxalate concentration.

## 1. Introduction

Primary hyperoxaluria (PH) is a genetic disorder of the glyoxylate metabolism in which patients have increased oxalate production, leading to renal damage caused by elevated concentrations of oxalate excreted via urine, which leads to the deposition of calcium oxalate crystals in the kidney [1]. Calcium oxalate (CaOx) includes calcium oxalate monohydrate (COM) and sub-stable calcium oxalate dihydrate (COD) [2]. Although COM has received far more attention than COD, it is undeniable that COD is the second most common crystal in urinary stones [3]. COD is a common calcium oxalate crystal found in the urine of patients with idiopathic urinary calcium stones [4]. Furthermore, studies have shown that COD stones are more likely to recur than COM stones [5]. Calcium oxalate crystals in patients with acute hyperoxaluria begin in the proximal tubule [6,7,8,9,10]. In general, proximal tubules have higher oxalate concentrations, and proximal tubule cells are more sensitive than distal tubule and collecting duct cells, they are more susceptible to damage from crystallization or by oxalates, and are more likely to secrete adhesion molecules [10]. Human renal proximal tubule epithelial cells (HK-2) can rapidly uptake the adherent CaOx crystals through pinocytosis and transfer to lysosomes inside the cells for degradation or dissolution [11,12,13], thereby removing residual crystals. Studies have shown that the health of cells directly affects the ability of cells to endocytose crystals; normal cells have a higher ability to endocytose crystals than damaged cells [14,15].

*Undaria pinnatifida* polysaccharide (UPP) has various pharmacological activities, such as antioxidant, immunomodulatory, and antitumor [16,17,18]. The polysaccharide (UPP-2) with a molecular weight of 1035.52 kDa isolated from *U. pinnatifida* was able to significantly promote the proliferation and cytocytosis of RAW264.7 cells and upregulate the mRNA expression of iNOS, TNF-α, IL-6, and IL-1β, and increase the secretion of nitric oxide, TNF-α, and IL-6, showing immunomodulatory functions [16]. Koh et al. [17] found that fucoidan isolated from New Zealand *U. pinnatifida* can inhibit the activities of starch hydrolase α-amylase, α-glucosidase, and amyloglucosidase, showing anti-diabetic properties. In addition, *U. pinnatifida* polysaccharide (SPUP) was able to inhibit the proliferation and migration rate of DU145 cells, and the expression of apoptosis-related factors caspase-3, caspase-9, and Bax was upregulated in the SPUP-treated group, which exhibited antitumor activity in prostate cancer [18]. Sulfation modification of plant polysaccharides can enhance many biological activities of polysaccharides, such as antioxidant, anti-inflammatory, and immunomodulatory [19,20,21,22,23]. Xie et al. [19] chemically modified *Cyclocarya paliurus* polysaccharides (CPs) by using the chlorosulfonic acid-pyridine method, (CSA-Pyr) and obtained four sulfated polysaccharides. Compared with the original polysaccharides, sulfated polysaccharides showed a better antioxidant activity. Yu et al. [23] modified cyanobacterial polysaccharides (CPs) to obtain two sulfated polysaccharides SCP3 and SCP5; CP, SCP3, and SCP5 all had immunomodulatory activities, CPs mainly stimulated the production of Th1-type cytokines, while SCP3 and SCP5 not only significantly enhanced the secretion level of Th1-type cytokines, but also promoted the secretion of Th2-type cytokines. Gunasekaran et al. [20] found that the sulfated-modified edible mushroom *Pleurotus eous* (Berk.) Sacc polysaccharides had stronger antioxidant and anticoagulant properties.

Sulfated-modified polysaccharides are primarily used for antioxidant, antibacterial, antiviral, antitumor, anticoagulation, and immunomodulatory research [24,25,26]. However, less research has been conducted on their use in repairing damaged cells [27,28]. Previously, four sulfated *U. pinnatifida* polysaccharides (UPPs) were synthesized and their structures were characterized. UPPs rich in acidic groups (–OSO_3_^−^) have good antioxidant activity and protect cells from oxidative damage induced by nano−COM [29].

In this study, we comparatively investigated the repair effect of four types of UPPs with different degrees of sulfation on damaged HK-2 cells and the difference in the ability of cells to endocytose nano−COD before and after polysaccharide repair, as well as the endocytic mechanism. It provides enlightenment for elucidating the mechanism of calcium oxalate crystal deposition caused by hyperoxaluria and for developing anti-stone drugs.

## 2. Experimental Part

### 2.1. Materials and Apparatus

Materials: Human renal proximal tubular epithelial cells (HK-2) were obtained from Shanghai Cell Bank, Chinese Academy of Sciences, China. Fetal bovine serum, cell culture medium (DMEM/F-12), and bovine serum albumin (BSA) were purchased from Gibco Biochemical Products Co., Ltd. (Beijing, China). Penicillin, streptomycin, pancreatin, phosphate-buffered saline (PBS), paraformaldehyde, fluorescein isothiocyanate (FITC), 2′,7′-dichlorofluorescein diacetate (DCFH-DA), 5,5′,6,6′-Tetrachloro-1,1′, 3,3′-tetraethyl-imidacarbocyanine iodide (JC-1) dye, Fluo-4 AM calcium ion fluorescent probe, and CCK-8 cell proliferation detection kit were purchased from Biyuntian Biotechnology Co. (Shanghai, China). 4,6-Diamidino-2-phenylindole (DAPI) staining was performed using an acridine orange (AO) kit, which was purchased from Solepipe Technology (Beijing, China). The malonaldehyde (MDA) kit and superoxide dismutase (SOD) kit were purchased from Jiancheng Institute of Biotechnology (Nanjing, China). MDC dye, lysosome red fluorescent probe (Lyso-Tracker Red), and Actin-Tracker Green were purchased from KeyGEN Biotech Co., Ltd. (Nanjing, China). Sulfur trioxide pyridine, formamide, and 3-aminopropyltriethoxysilane (AMPTES) were purchased from Shanghai Aladdin Biochemical Technology Co., Ltd. (Shanghai, China). Oxalate and other chemical reagents were of analytical grade, which were purchased from China Guangzhou Chemical Reagent Factory (Guangzhou, China).

The *U. pinnatifida* polysaccharide (UPP0) with a molecular weight of 8.33 kDa was purchased from Ciyuan Biotechnology Co. in Shaanxi, China, and was purified in accordance with our previous study [29]. The characterization results showed that the UPP0 molecule is a block of sequentially linked residues of fucose and galactose. Four sulfated polysaccharides with sulfate group (–OSO_3_^−^) contents of 1.59% (UPP0), 6.03% (UPP1), 20.83% (UPP2), and 36.39% (UPP3) were obtained by using a sulfur trioxide–pyridine method. Fourier transform infrared spectrometry (FT-IR), ^1^H NMR, and ^13^C NMR studies showed that the hydroxyl groups at the C2 and C4 positions of the 1,3-linked Fucp residues of UPP0 and the C3 and C6 positions of the β-D-galactopyranose residues were replaced by –OSO_3_^−^ groups, indicating the successful sulfation modification of UPP0 [29].

Nano-calcium oxalate dihydrate (nano−COD) crystals with a size of approximately 100 nm were synthesized in accordance with our previous study [30], and the characterization showed that it was the target product.

Apparatus: An optical microscope (OLYMPUS, CKX41, Tokyo, Japan), FT-IR (EQUINOX 55, Bruker, Ettlingen, Germany), microplate reader (Safire2, Tecan, Männedorf, Switzerland), laser confocal microscope (LSM510 META DuoScan, Carl Zeiss Meditec AG, Berlin, Germany), inverted fluorescence microscope (Leica DMRA2, Wetzlar, Germany), and flow cytometer (FACS Aria, BD Corporation, San Diego, CA, USA) were used in the study.

### 2.2. Experimental Methods

#### 2.2.1. Cell Culture and Establishment of an Injury Model

(1). Cell culture

HK-2 cells were cultured in DMEM-F12 containing 10% FBS, 100 U/mL of penicillin, and 100 μg/mL of streptomycin at 37 °C and 5% CO_2_ in saturated humidity. The cells were passaged by trypsin digestion. All cells in the experiment were cultured in seed plates for 24 h to achieve 80% confluence before treatment;

(2). Construction of a damage model

The cell density in the well plate or confocal dish was 1.0 × 10^5^ cells/mL, and 0.1–2 mL/well of cell suspension was added on the basis of the size of the well plate or confocal dish, and incubated for 24 h. The cells were incubated using a serum-free DMEM-F12 medium for 12 h to synchronize the cells. Then, the medium was aspirated, and the cells were washed two times with PBS.

Cell experimental models were divided into three groups:

(A). Normal control group: cultured in a serum-free medium;
(B). Damage control group: the cells were injured with 2.8 mmol/L of oxalate solution for 3 h, removed by suction, and incubated with a serum-free medium for 12 h;
(C). Polysaccharide repair group: after the cells were injured by 2.8 mmol/L of oxalate solution for 3 h, they were removed by suction and added to 60 μg/mL of UPP serum-free medium for repair and culture for 12 h.

#### 2.2.2. Cytotoxicity of UPPs and Changes in Cell Viability, SOD, and MDA before and after UPP Repair

(1). Cytotoxicity assay

The cell seed plate was the same as that described in Section 2.2.1. Cytotoxicity experiments were divided into two groups: (1) normal control group and (2) polysaccharide group. In addition, 20, 40, 60, and 80 μg/mL of UPP solutions were set for 12 h. Upon reaching the action time, 10 μL of CCK-8 reagent was added to each well and incubated for 2 h. The cell viability was calculated by measuring the OD value at 450 nm using a microplate reader, and three parallel groups were set for each condition;

(2). Cell viability assay

The cell seed plate and experimental groups were the same as those described in Section 2.2.1. The polysaccharide concentration of the polysaccharide repair group was set to 40, 60, and 80 μg/mL. Upon reaching the repair time, the assay was performed in accordance with the method of the CCK-8 kit;

(3). SOD and MDA level detection

The cell seed plate and experimental groups were the same as those described in Section 2.2.1. Upon reaching the repair time, detection was performed in accordance with the instructions of the SOD kit and MDA kit.

#### 2.2.3. Cell Morphology and Cytoskeleton Observation

(1). Observation of cell morphology

The cell seed plate and experimental groups were the same as those described in Section 2.2.1. Upon reaching the repair time, the cells were washed with PBS. The cell morphology was directly observed under an optical microscope;

(2). Cytoskeleton observation

The cell seed plate and experimental groups were the same as those shown in Section 2.2.1. Upon reaching the repair time, the cells were washed with PBS and then fixed with 3.7% paraformaldehyde solution at room temperature for 20 min. Then, Actin-Tracker Green was diluted 1:100 with PBS containing 5% BSA and 0.1% Triton X-100, and 600 μL of Actin-Tracker Green staining working solution was added dropwise to the confocal dish in accordance with each sample and incubated at room temperature for 60 min in the dark. Then, the cells were washed 2–4 times with PBS and 0.1% Triton X-100, for about 5 min each time. Subsequent observations were performed using confocal fluorescence microscopy.

#### 2.2.4. Observation of the Cell Healing Rate

The experimental treatment was conducted as described in Section 2.2.1, to reach the repair time using 10 μL of the tip in the cell culture line, it was marked. The dropped cells were removed by washing with PBS and then a new medium was added at 0, 12, and 24 h to observe scratch spacing, and photos were taken to record and calculate the healing rate [31].

#### 2.2.5. Detection of the ROS Expression and Mitochondrial Membrane Potential

The experimental treatment was performed as described in Section 2.2.1. Once the repair was completed, the medium was removed by suction and washed two times with PBS.

(1) ROS detection: 500 μL of DCFH-DA diluted at a ratio of 1:1000 was added, incubated at 37 °C for 30 min, and observed under a fluorescence microscope. Fluorescence images were analyzed using ImageJ for the semi-quantitative fluorescence analysis;
(2) Mitochondrial membrane potential detection: 5 μg/mL of JC-1 working solution was added, stained at 37 °C for 1 h, washed with PBS three times, and observed using an inverted fluorescence microscope. Fluorescence images were analyzed using ImageJ for the semi-quantitative fluorescence analysis [29].

#### 2.2.6. Detection of Intracellular Ca^2+^, Lysosomal Integrity and Autophagy

The experimental treatment was performed as described in Section 2.2.1. Once the repair was completed, the medium was removed by suction and washed two times with PBS.

(1). Detection of intracellular Ca^2+^: Cells were incubated with 2.5 μM Fluo-4 AM for 45 min in the dark, washed with PBS, fixed with 3.7% paraformaldehyde, and then stained with DAPI, and this was followed by direct observation using a confocal fluorescence microscope;
(2). Detection of lysosomal integrity: Cells were stained with 5 μg/mL of AO working solution for 15 min, and then observed using an inverted fluorescence microscope after washing with PBS. Fluorescence images were analyzed semi-quantitatively by ImageJ;
(3). Autophagy detection: Cells were washed two times with 1 × wash buffer, and 1 mL of 10% monodansylcadaverine (MDC) staining solution was added into each well and incubated in an incubator for 45 min in the dark. Once washed two times with a wash buffer, observation was made under a fluorescence microscope. Finally, ImageJ was used for the semi-quantitative fluorescence analysis of MDC.

#### 2.2.7. Qualitative Observation and Quantitative Detection of Nano−COD Endocytosis

(1). FITC fluorescent labeling and characterization of nano−COD: Based on the experimental method of our previous study [29], nano−COD was prepared using a two-step reaction.

AMPTES (5 mL) was reacted with 0.05 g of COD in 50 mL of absolute ethanol for 3 h at 74 °C with stirring. Then, 0.025 g of FITC was added, and the reaction was kept for 6 h. Subsequently, the reaction was washed several times with anhydrous ethanol and deionized water until it was free of FITC. Finally, the labeled nanoparticles (FITC−COD) were dried. In addition, an appropriate amount of crystals were selected using a fluorescence microscope, and the proportion of stained crystals was detected by flow cytometry;

(2). Qualitative observation of endocytosis: The experimental treatment was the same as that described in Section 2.2.1. Once the repair was completed, the medium was aspirated and washed two times with PBS. Then, 200 μg/mL of the freshly prepared FITC-labeled COD was added to the medium and incubated for 6 h. Then, Lyso-Tracker Red (800 μL) was added to label the lysosomes for 2 h. The cells were fixed with paraformaldehyde, and the cell nuclei were stained with DAPI. The crystal distribution was observed under a confocal microscope;
(3). Quantitative detection of endocytosis: The experimental treatment was the same as that described in Section 2.2.1. Once the repair was completed, the medium was aspirated and washed two times with PBS. Then, 200 μg/mL of the freshly prepared FITC-labeled COD was added and incubated for 6 h. The cells were treated with 5 mM EDTA for 5 min to remove the adhered crystals and collected, and the proportion of fluorescent cells was detected by flow cytometry.

#### 2.2.8. Statistical Analysis

Experimental data were expressed as the mean ± standard deviation ($\overset{-}{x}$ ± SD). The experimental results were statistically analyzed using SPSS 13.0 (SPSS Inc., Chicago, IL, USA). Multiple-group comparisons were performed using one-way ANOVA, followed by the Tukey post hoc test. *p* < 0.05 indicated a significant difference; *p* < 0.01 indicated an extremely significant difference; *p* > 0.05 indicated no significant difference.

## 3. Results

### 3.1. UPPs Are Non-Cytotoxic, Which Can Improve the Viability of Injured Cells

(1). UPPs are not cytotoxic

**Figure 1:**
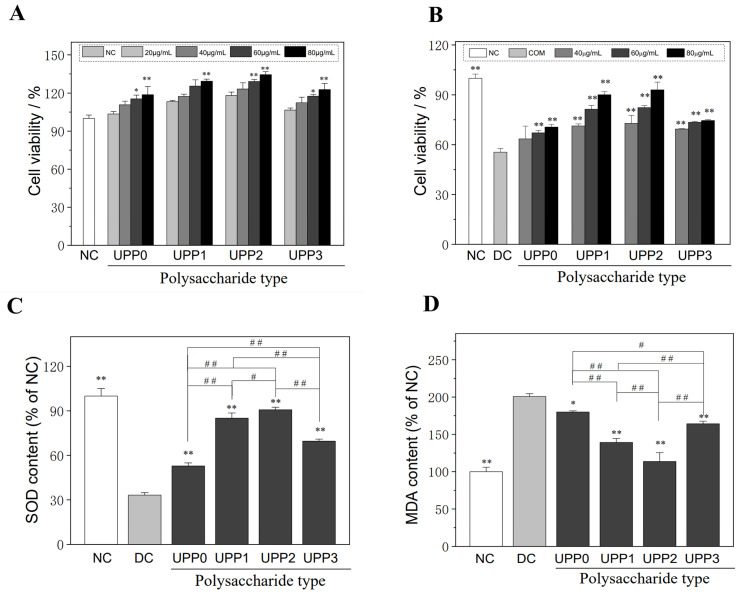
Cytotoxicity of UPPs (**A**) and changes in cell viability (**B**), intracellular SOD (**C**), and MDA (**D**) levels of UPPs before and after repair of damaged HK-2 cells. NC: normal control group, DC: damage control group, injured with 2.8 mmol/L oxalate solution for 3 h; polysaccharide concentration: 60 μg/mL; repair time: 12 h. * *p* < 0.05, ** *p* < 0.01 compared with DC group; # *p* <0.05, ## *p* < 0.01 compared between the different UPP treatment groups.

As shown in Figure 1A, after co-incubating HK-2 cells with different concentrations of sulfated *U. pinnatifida* polysaccharides (UPP0, UPP1, UPP2, and UPP3), their sulfate group (–OSO_3_^–^) contents were 1.59%, 6.03%, 20.83%, and 36.39%, respectively, for 12 h. The cell viability ranged from 103.50% to 134.43%, which were higher than those of the normal control group (100.00%). Therefore, UPPs had no toxicity to HK-2 cells in the concentration range of 20–80 μg/mL, which promoted cell growth;

(2). UPPs can improve the viability of damaged cells

UPPs had a repairing effect on HK-2 cells damaged by oxalate (Figure 1B). Once they were repaired using different concentrations of UPPs, the cell viability increased from 55.47% in the damaged group to 63.51–92.99%. At the same concentration, the repairing effect of UPPs was as follows: UPP0 < UPP3 < UPP1 < UPP2. That is, UPP2 with medium –OSO_3_^−^ group content (20.83%) had the best repair effect, and the –OSO_3_^−^ group content was too high or too low to reduce its repair ability. For the same polysaccharide, the cell viability was positively correlated with a polysaccharide concentration in the range of less than 80 μg/mL, indicating a concentration effect. For example, when the UPP2 concentration was increased from 40 to 80 μg/mL, the cell viability increased from 72.81% ± 4.91% to 92.99% ± 4.71%.

### 3.2. UPPs Improve the Cellular Antioxidant Capacity and Reduce Lipid Peroxidative Damage

(1). UPPs increase SOD expression

SOD is the common antioxidant enzyme in cells, which can scavenge intracellular superoxide anion, thereby reducing cellular and oxidative damage [32]. Following oxalate solution injury, the SOD activity of HK-2 cells decreased to 33.20% ± 1.65% of the control value (Figure 1C). Once repaired with 60 μg/mL of UPPs, the SOD activity was greatly improved (52.82–90.76%), and the UPP2 group reached 90.76% ± 1.70% of the normal control group.

(2). UPPs reduce the malondialdehyde (MDA) levels

The MDA level is an important indicator reflecting lipid peroxidative damage [33]. As shown in Figure 1D, the amount of MDA released by cells in the damage group increased by 200.83% ± 3.67% that of the normal control group. Once repaired using 60 μg/mL of each UPP, the level of MDA was significantly decreased (179.94–113.65%), indicating that UPPs could reduce the degree of cellular lipid peroxidative damage, and UPP2 had the highest activity (113.65% ± 11.78%).

### 3.3. UPPs Improve Cell Morphology and Cytoskeleton

(1). UPPs improve cell morphology

**Figure 2:**
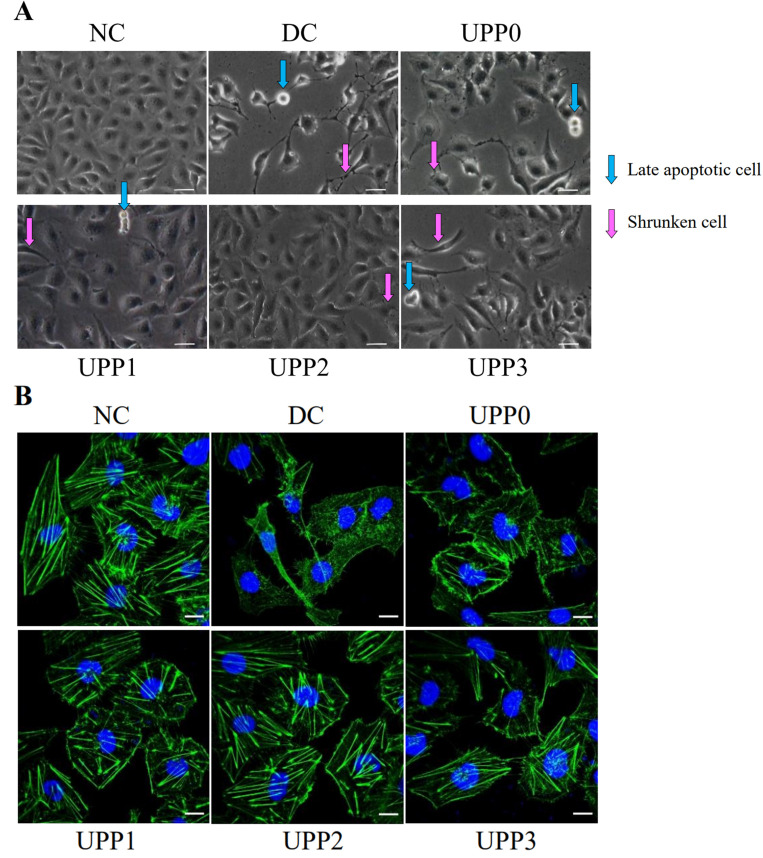
Differences in cell morphology (**A**) and cytoskeleton (**B**) before and after UPPs repaired the damaged HK-2 cells. NC: normal control group, DC: damage control group, injured with 2.8 mmol/L oxalate solution for 3 h; polysaccharide concentration: 60 μg/mL; repair time: 12 h. Scale bar: (**A**) 50 µm, (**B**) 10 µm.

Microscopic observation showed that normal control cells were round and plump, whereas the cells in the damage group shrank severely. Once the cells in the damage group were repaired by various UPPs, the cell morphology was improved to varying degrees, and the morphology was more plump and regular (Figure 2A);

(2). UPPs improve the cytoskeleton

The cytoskeleton was fluorescently labeled by Actin-Tracker Green, and changes in the cytoskeleton before and after repair were observed (Figure 2B). In the normal control group, the cytoskeleton was complete and clear, whereas the outline and skeleton of the cells in the damage group became blurred. Compared with the damage group, the cells of each UPP repair group were significantly improved, and the UPP2 group showed remarkably improved cell morphology and skeleton.

### 3.4. UPPs Promote the Healing of Injured Cells

**Figure 3:**
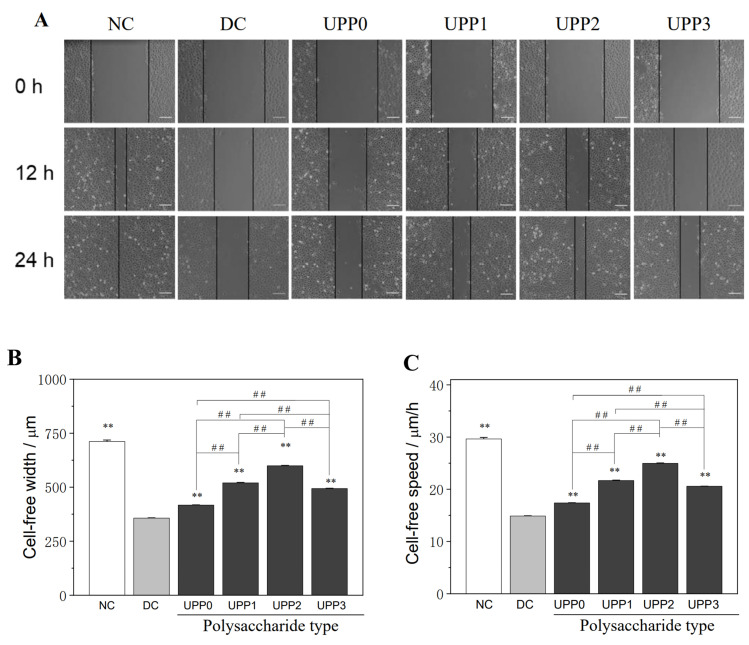
Changes in the cell healing ability of UPPs before and after the repair of damaged HK-2 cells. (**A**) Cell healing observed by ordinary microscope; (**B**) histogram of cell healing width within 24 h; (**C**) histogram of cell healing rate within 24 h. ** *p* < 0.01 compared with the DC group; ## *p* <0.01 compared between the different UPP treatment groups. Scale bar: 150 µm.

The healing of cells in each group at 0, 12, and 24 h was observed by scratch experiments (Figure 3). The scratches of the cells in the normal control group were healed after 24 h, and the healing rate (υ) was 29.64 ± 0.29 μm·h^−1^. The cells in the damage group healed the slowest, υ = 14.87 ± 0.06 μm·h^−1^. The healing rate of the UPP repair group was faster than that of the damage group, and υ of UPP0, UPP1, UPP2, and UPP3 groups was 17.38 ± 0.05, 21.67 ± 0.09, 24.97 ± 0.08, and 20.58 ± 0.02 μm·h^−1^, respectively.

### 3.5. UPPs Reduce ROS Production and Increase Intracellular Mitochondrial Membrane Potential (Δψm)

(1). Reduced intracellular ROS production

**Figure 4:**
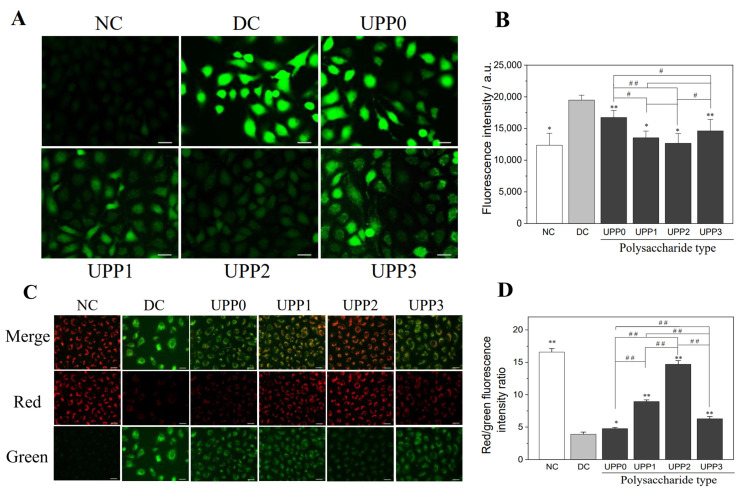
Fluorescence intensity graph (**A**) and semi-quantitative histogram (**B**) of ROS release by UPPs before and after the repair of damaged HK-2 cells, and fluorescence intensity graph (**C**) and semi-quantitative histogram (**D**) of mitochondrial membrane potential. NC: normal control group, DC: damage control group, injured with 2.8 mmol/L oxalate solution for 3 h; polysaccharide concentration: 60 μg/mL; repair time: 12 h. Scale bar: 150 µm. * *p* < 0.05, ** *p* < 0.01 compared with the DC group; # *p* <0.05, ## *p* <0.01 compared between the different UPP treatment groups.

As shown in Figure 4A, the cells in the normal control group showed weak green fluorescence, whereas the cells in the damage group expressed significantly enhanced fluorescence, indicating that the ROS in the damage group was significantly increased. Once the cells in the damage group were repaired by UPPs, the level of ROS decreased; the fluorescence semi-quantitative results showed that UPP2 had the strongest ability to scavenge ROS production (Figure 4B);

(2). Increased intracellular Δψm

Decreased Δψm is an important marker of cellular oxidative damage. When Δψm decreased, the fluorescent probe molecule JC-1 switched from red fluorescence (aggregates) to green fluorescence (monomers). As shown in Figure 4C, control cells exhibited bright red fluorescence, that is, high Δψm. The cells in the damage group primarily showed green fluorescence, indicating that oxalate solution injury caused a significant decrease in Δψm of HK-2 cells. Once repaired by UPPs, red fluorescence gradually increased, indicating an increase in Δψm, among which Δψm in the UPP2 group was the closest to the normal control group (Figure 4D).

### 3.6. UPPs Reduce the Intracellular Ca^2+^ Concentration

**Figure 5:**
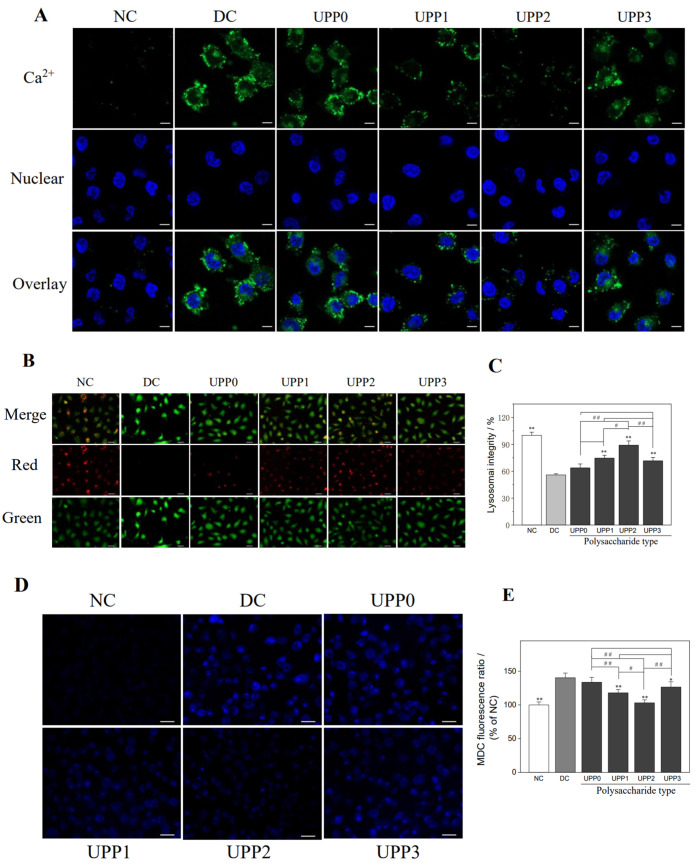
Changes of the intracellular Ca^2+^ level (**A**), lysosomal integrity (**B**,**C**), and autophagy activity (**D**,**E**) before and after the repair of damaged HK-2 cells by UPPs. Intracellular Ca^2+^ was stained with Fluo-4 AM (red-green fluorescence); nuclei were stained with DAPI (blue fluorescence). NC: normal control group, DC: damage control group, injured with 2.8 mmol/L oxalate solution for 3 h; polysaccharide concentration: 60 μg/mL; repair time: 12 h. Scale bar: (**A**) 10 µm, (**B**) and (**D**)50 µm. * *p* < 0.05, ** *p* < 0.01 compared with DC group; # *p* <0.05, ## *p* <0.01 compared between the different UPP treatment groups.

The increased concentration of free intracellular Ca^2+^ ions may disrupt mitochondrial membrane potential, thereby leading to apoptosis and necrosis. The fluorescent probe Fluo-4 AM can pass through the cell membrane and be converted into Fluo-4 by intracellular esterase. Then, it specifically binds to Ca^2+^ ions to generate a substance with strong green fluorescence. As shown in Figure 5A, the green fluorescence of the cells in the normal control group was weak, whereas the fluorescence intensity of the damaged cells was significantly enhanced, indicating that the intracellular Ca^2+^ ion concentration in the damage group was significantly increased. The Ca^2+^ ion concentration of each UPP repair group was between the normal control group and damage group, and the UPP2 repair group decreased the most Ca^2+^ ion.

### 3.7. UPPs Improve the Lysosomal Integrity

Acridine orange (AO) can bind to acid hydrolase in lysosomes and emit orange–red fluorescence, whereas it emits green fluorescence in the cytoplasm [34]. As shown in Figure 5B, the lysosomal structure of the normal control group was relatively complete, and the superposition of red fluorescence and green fluorescence showed more orange–red fluorescence. However, the red fluorescence of cells in the damage group were significantly reduced, and the integrity of lysosomes dropped to 55.92% (Figure 5C). Once repaired by UPPs, the integrity of lysosomes increased to 63.74–89.01%, of which UPP2 showed the best repair effect.

### 3.8. UPPs Reduce Autophagic Activity

Monodansylcadaverine (MDC) is an autofluorescent dye that can be used to label autophagic vacuoles, and the accumulation of MDC-labeled vesicles indicates increased autophagic activity [35,36]. The fluorescence intensity of autophagy before and after the repair of damaged HK-2 cells by each UPP was observed by fluorescence microscopy (Figure 5D). Only faint blue fluorescent spots were observed in the normal control group, whereas a large number of autophagic vacuoles, that is, diffused blue fluorescent spots, appeared in the damage group, indicating that cell damage promoted autophagic activity. Once the damaged cells were repaired by UPPs, the blue fluorescence intensity decreased, that is, the autophagic vacuoles were significantly reduced, and the blue fluorescence intensity of the UPP2 group was close to that of the normal control group (Figure 5E). Therefore, all UPPs could inhibit the autophagic activity of cells, and UPP2 had the best inhibitory effect.

### 3.9. UPP Repair Promotes Cellular Endocytosis of Nano−COD

(1). Synthesis, fluorescent labeling, and characterization of nano−COD crystals

**Figure 6:**
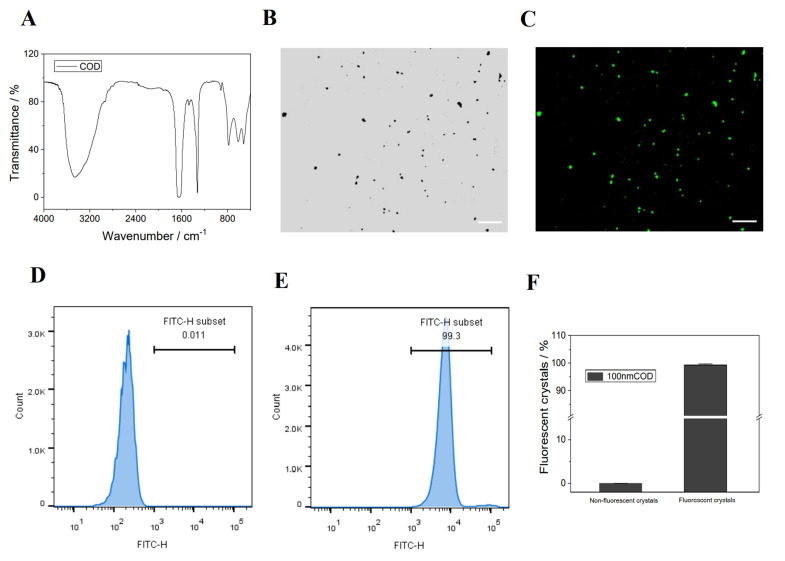
Fluorescent labeling and characterization of nano−COD crystals (**A**) FT-IR spectrum of the nano−COD crystal; (**B**,**C**) white light and fluorescence microscopy images of FITC−COD crystals; (**D**–**F**) flow cytometry graphs and statistical histograms of nano−COD crystals before and after FITC labeling. Scale bar: 50 µm.

The FT−IR spectrum (Figure 6A) shows a broad absorption peak at 3490–3059 cm^−1^. The carbonyl asymmetric stretching vibration υas(COO^−^) appears at 1647 cm^−1^, and the symmetrical stretching vibration υs(COO^−^) appears at 1324 cm^−1^. The absorption bands of the crystal in the fingerprint region appear at 916 and 615 cm^−1^. These results indicate that the synthesized crystals are the target crystalline COD [37].

Once the nano−COD was fluorescently labeled FITC, FITC−COD with green fluorescence was obtained. The results of fluorescence microscopy and flow cytometry showed that the nano−COD had been successfully labeled (Figure 6B–F).

(2). Qualitative and quantitative detection of nano−COD endocytosis by cells

**Figure 7:**
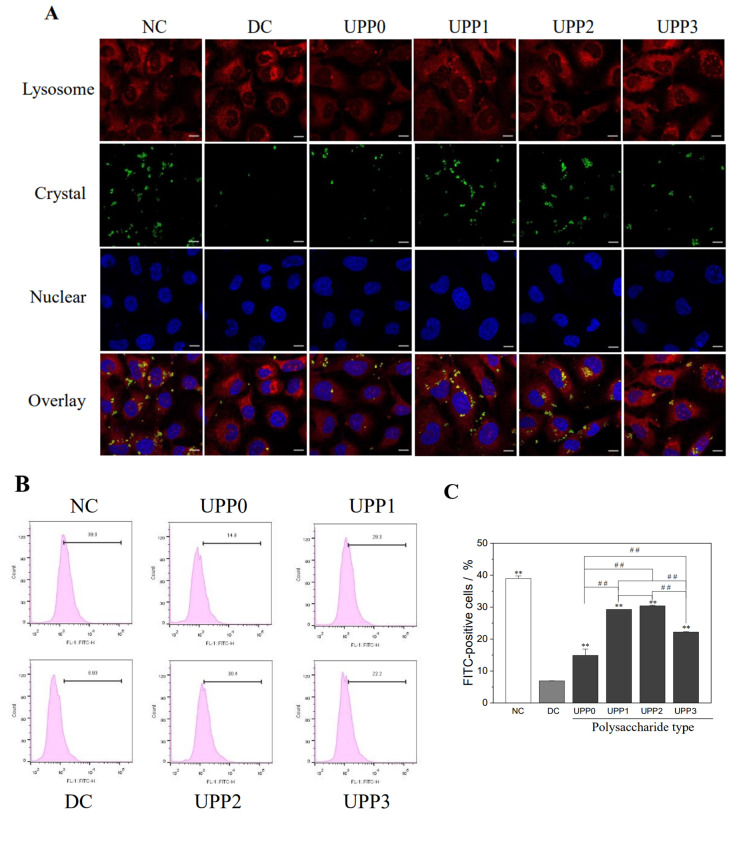
Changes in the endocytosis of nano−COD in HK-2 cells before and after UPP repair. (**A**) Qualitative observation using confocal microscope; (**B**) scatter plot of the quantitative flow cytometry; (**C**) quantitative histogram of endocytosis. Cells were treated with FITC-labeled 200 μg/mL COD crystals (green fluorescence) for 6 h; crystal concentration: 200 μg/mL; endocytosis time: 6 h. Lysosomes were stained with Lyso-Tracker Red (red fluorescence); nuclei were stained with DAPI (blue fluorescence). Scale bar: 10 µm. ** *p* < 0.01 compared with the DC group; ## *p* <0.01 compared between the different UPP treatment groups.

Lysosomes, nuclei, and COD crystals were fluorescently labeled with Lyso-Tracker Red, DAPI, and FITC, respectively, and the intracellular distribution of endocytosed nano−COD was observed by confocal microscopy (Figure 7A). The amount of endocytosed nano−COD crystals in the normal control group was significantly higher than that in the damage group, and the nano−COD crystals were primarily located in lysosomes. Once the damaged cells were repaired by UPPs, the ability of endocytosed crystals was enhanced, and the UPP2 group with moderate molecular weight had the greatest increase in the amount of endocytosed crystals.

Figure 7B shows the quantitative detection of the endocytosis of nano−COD crystals by HK-2 cells before and after UPP repair by flow cytometry. Following the removal of adherent crystals by EDTA complexation, FITC−positive cells contained endocytic crystals [38]. The proportion of FITC-positive cells in the normal control group was 39.0%, and that in the damage group decreased to 6.93% (*p* < 0.01). Following the UPP repair, the endocytosis of nano−COD crystals gradually increased (14.9–30.4%), and the UPP2 group increased the most (30.4%).

## 4. Discussion

### 4.1. Sulfated Polysaccharides Can Repair Damaged Cells

For normal organisms, ROS maintain a dynamic balance at a certain level. When body cells are damaged and the required antioxidants (such as antioxidant enzymes) cannot be produced normally, a large amount of ROS begins to accumulate in the cells, destroying the structure of organelles and biomolecules, thereby affecting the normal function of cells and leading to cell damage and death [39,40]. Studies have shown that reducing excess ROS production reduces kidney damage and deposition of CaOx crystals in the kidney, thereby reducing the risk of lithogenesis [41,42].

In the present study, polysaccharides effectively scavenge excess ROS, and the scavenging effect of sulfated UPPs was better than that of unsulfated UPP0 (Figure 4A). Many studies have shown similar patterns [43,44,45]. Han et al. [43] modified the sulfated polysaccharide of CPP0.05 by using the chlorosulfonic acid-pyridine (CSA-Pyr) method, which could better scavenge DPPH, ABTS, and hydroxyl radicals through the Keap1-Nrf2 pathway. Oxidative stress can also be inhibited by improving the enzymatic activities of SOD and CAT in dendritic cells in the presence of H_2_O_2_. Zhao et al. [44] obtained sulfated polysaccharide *Potentilla anserina* L polysaccharide (SPAP) by using the method of Han, which has a better scavenging ability of DPPH, hydroxyl radicals, superoxide radicals; chelating capacity of Fe^2+^ ions and reducing ability. Li et al. [45] demonstrated that sulfated modified alfalfa polysaccharides and their antioxidant and antibacterial properties, that promote the proliferation of bovine intestinal epithelial cells and inhibit obesity, were improved.

Among various polysaccharide derivatives, sulfated polysaccharide is a stronger antioxidant because of the following reasons:

(1). The introduction of –OSO_3_^−^ groups changes the configuration and orientation of polysaccharides, making it easier to expose hydroxyl groups in aqueous solution [46];
(2). The strong acidic environment generated by the introduction of –OSO_3_^−^ substituents into sulfated polysaccharide molecules leads to weaker dissociation energy of hydrogen bonds, thereby increasing the hydrogen-donating ability of polysaccharide derivatives [47];
(3). The introduction of –OSO_3_^−^ activates the hydrogen atoms of the anomeric carbon and increases the hydrogen-donating capacity [43].

The abovementioned factors provide strong theoretical support for the effective removal of excess ROS by polysaccharides. However, to date, the underlying exact mechanism remains unclear because the relationship between the antioxidant activity of polysaccharides and the physicochemical properties of polysaccharides or the structural characteristics of polysaccharides have not been fully elucidated and confirmed, which also require further in-depth exploration by researchers.

Mitochondria play a key role in maintaining normal cell function, and their damage is a major cause of tissue damage caused by energy stress and ROS overproduction [48,49]. Lysosomes are the major degradative organelles in cells, linking endocytosis and autophagy pathways [50]. Therefore, the normal function of mitochondria and lysosomes is crucial for the body. Mulay et al. [51] showed that calcium oxalate, monosodium urate, or calcium pyrophosphate dihydrate crystals can cause cell damage, trigger mitochondrial permeability transition and lysosomal cathepsin leakage, and increase ROS release. Park et al. [52] showed that transcription factor EB (TFEB) is activated through lysosomal Ca^2+^ release and endoplasmic reticulum (ER) → lysosomal Ca^2+^ refill, and TFEB-dependent mitophagy is transactivated by mitophagy receptors, such as Ndp52 or Optn, thereby improving mitochondrial function that plays an important role in the adaptation of pancreatic β cells to metabolic stress.

Renal epithelial cell damage may be important in the formation of stones in conditions of hyperoxaluria [53]. When cells are damaged, the mitochondria overproduce ROS, which oxidatively damages mitochondria and other organelles, thereby increasing the ROS release (Figure 4A), and this negative feedback exacerbates cell damage or even death. The addition of UPPs can not only improve the morphology of damaged cells (Figure 2), promote cell proliferation and healing (Figure 1B and Figure 3), remove excess ROS (Figure 4A), and block this vicious cycle, thereby reducing mitochondria and lysosomal damage (Figure 4C and Figure 5B), but also increase the release of intracellular SOD (Figure 1C) and decrease the production of MDA (Figure 1D) in response to the oxidative stress process. Intracellular Ca^2+^ levels are also critical for maintaining cellular homeostasis. Cellular injury led to the efflux of Ca^2+^ ions from the organelles, resulting in increased intracellular Ca^2+^ levels (Figure 5A), which in turn stimulated the production of ROS in mitochondria. Therefore, maintaining the stability of intracellular Ca^2+^ and the normal function of organelles is important [52,54,55]. In addition, UPPs did not express toxicity to HK-2 cells, but they promoted cell proliferation (Figure 1A). UPPs can also remove excess ROS produced by damaged cells and resist further oxidative damage of ROS on cells by increasing the activity of antioxidant enzymes (Figure 1B).

Autophagy, as a cellular self-protection mechanism, can remove damaged subcellular organelles and foreign bodies. The organelles in the damage group were severely damaged, and autophagy was significantly enhanced to restore normal state and function (Figure 5D). Following the addition of UPPs, the cell state and function were restored, and the autophagic activity was reduced. Shen et al. [56] found that cadmium increased ROS production to induce autophagic injury in chicken embryo fibroblasts (CEFs). In addition, Astragalus polysaccharide (APS) can significantly reduce ROS production and expression of LC3-II and Beclin-1 proteins, increase mTOR expression and antioxidant level, restore morphological damage caused by cadmium exposure, and alleviate cadmium-induced autophagy damage in CEFs. *Porphyra yezoensis* and Astragalus polysaccharides can significantly reduce ROS production, restore mitochondrial membrane potential, reduce excessive apoptosis and autophagy, upregulate α-smooth muscle actin expression, inhibit alkaline phosphatase activity, and reduce calcium deposition on the surface of A7r5 cells [57]. Li et al. demonstrated that *P. sanguineus* polysaccharide (PPS) can delay the pathological changes of inflammatory bowel disease by upregulating the expression of ZO-1 and E-cadherin, proliferating cell nuclear antigen (PCNA), and reducing the proportion of Th cells to inhibit the helper Th cell-mediated immune response. Moreover, it reduces the activity of myeloperoxidase (MPO) and the release of several interleukins and chemokines in the colon, indicating its significant inhibitory effect on autophagy [58].

**Figure 8:**
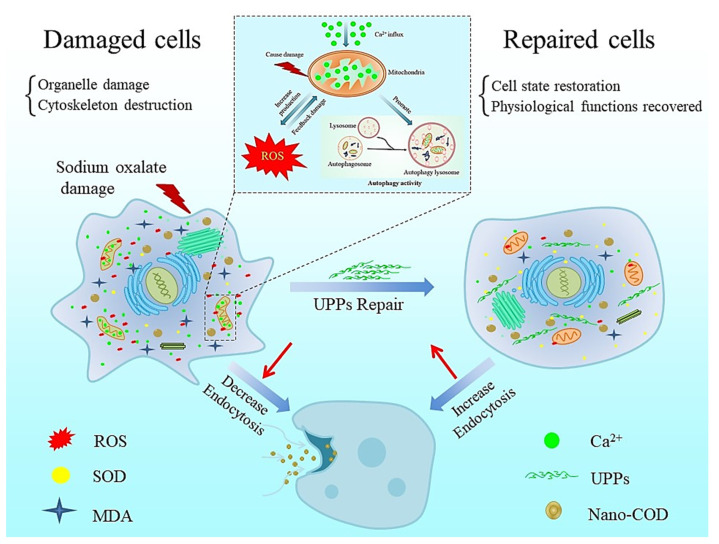
The endocytosis mechanism of nano−COD crystals before and after the UPPs repair the oxalate-induced oxidative damage in HK-2 cells. The dashed box shows the negative feedback regulation of ROS on mitochondria and the potential relationship between increased mitochondrial damage leading to increased autophagic activity.

High concentrations of oxalate mediate the damage of intracellular organelles (lysosomes and mitochondria), leading to the influx of Ca^2+^ ions in the organelles (lysosomes and ER) into the cytoplasm, which together with the ROS released from mitochondrial damage further directs the massive accumulation of mitochondrial ROS [55], thereby triggering a cellular self-protection mechanism that increases autophagic activity in cells to remove damaged organelles and foreign bodies. The possible mechanism is shown in the dashed box above in Figure 8.

At present, many studies have been conducted on polysaccharides, which improve diseases by repairing damaged cells. For example, Li et al. [59] showed that food-derived natural polysaccharides can effectively prevent and improve intestinal mucosal damage and immune system diseases caused by CTX. Ying et al. [60] extracted three polysaccharide components from Liubao tea, that showed a good repair effect on damaged human umbilical vascular endothelial cells. Our previous study has found that laver polysaccharides with different molecular weights showed good repairing efficacy on oxalate solution–injured renal epithelial cells [54].

In this study, sulfated-modified UPPs could eliminate excess intracellular ROS, inhibit the overproduction of ROS, protect organelles (mitochondria, lysosomes, and cytoskeleton) from oxidative damage, and increase the production of antioxidant enzymes. The inhibition of oxidative stress can produce harmful substances to achieve the repair of the cell state and function. Therefore, the sulfated polysaccharide UPP2 has great application potential in repairing cells and inhibiting or delaying the formation of calcium oxalate crystals.

### 4.2. Reasons for the High Activity of UPPs with Moderate –OSO_3_^−^ Content

The molecular weight of polysaccharides, the type of sugar backbone, and acidic functional groups affect the biological activity of polysaccharides [61]. Molecular weight has been widely demonstrated as an important factor affecting the biological activity of polysaccharides [62,63,64]. In general, low-molecular-weight polysaccharides have better biological activity [63,64]. The content of acidic functional groups is another important parameter affecting polysaccharides. Theoretically, as the content of sulfate groups increases, the biological activity of polysaccharides will gradually increase. However, in this study, UPP2 with a moderate –OSO_3_^−^ group had the best pharmacological activity, and a too high or too low –OSO_3_^−^ group content reduced its biological activity probably because of the combined effect of –OSO_3_^−^ group content and other factors [62,65]. Wu et al. [62] showed that the antioxidant and hypoglycemic activities of okra pectin polysaccharide were related to its molecular weight and esterification degree, and its immunomodulatory activity was closely related to its branching degree, molecular weight, and esterification degree. In this study, the molecular weight of the polysaccharide is likely to be the second influencing factor because the molecular weight of the polysaccharide gradually increases with the increase of the sulfation degree, that is, the molecular weight of UPP3 is the heaviest. The heavy molecular weight leads to large steric hindrance, that hinders the exposure of active groups and the entry of polysaccharides into cells to exert pharmacological effects [66]. In addition, the study by Zuo et al. demonstrated that low-molecular-weight polysaccharides likely penetrate cells [67]. In this study, the best restorative efficacy of UPP2 was attributed to its higher sulfate group content and lower molecular weight.

### 4.3. UPPs Promote Crystal Endocytosis

Considering that the conditions in the human body are complex and the organic components in urine of different patients are different, chemically synthesized nano−COD crystals were adopted for in vitro simulation. In this study, UPPs were used to repair oxidatively damaged HK-2 cells, and the effect of cell repair on COD crystal endocytosis was explored. The endocytosis of COD by HK-2 cells was closely related to the state of the relevant subcellular organelles. Crystal endocytosis is an active transport mechanism involving actin cytoskeleton-mediated macropinocytosis, and actin cytoskeleton disorders or assembly defects will weaken crystal endocytosis [12]. Therefore, the proper functioning of the actin cytoskeleton is crucial. Second, the endocytosis of cells requires energy, in which mitochondria, as the ATP production workshop, plays an important role. Third, after the endocytosis of foreign matter, it should be decomposed by lysosomes, which act as the digestion workshop; thus, the normal function of lysosomes is indispensable.

When the cells are damaged, the intracellular cleaner (lysosomes) and energy-supplying organelle (mitochondria) are damaged to varying degrees, and the cytoskeleton is damaged. The cell is in poor condition, resulting in weak endocytosis. Once repaired by UPPs, the mitochondrial membrane potential of the damaged cells recovered to close to normal cellular levels (Figure 4C). Lysosomal integrity was also improved (Figure 5B), and the cytoskeleton was restored (Figure 2B), thereby providing conditions for endocytic crystals. Therefore, the amount of endocytosed crystals in the UPP repair group increased, particularly the UPP2 repair group, because of its best cell state. The amount of crystal endocytosis was the highest, which was close to the normal control group (Figure 7). Therefore, UPPs can improve the ability of cells to take up nano−COD by repairing oxidatively damaged renal epithelial cells, including repairing mitochondria, lysosomes, and restoring the cytoskeleton, thereby reducing the risk of stone formation. The mechanism is shown in Figure 8.

Under normal circumstances, renal tubular cells internalize the formed CaOx crystals into the cell through macropinocytosis and decompose into Ca^2+^ and Ox^2−^ under the action of lysosomal hydrolase [13,15]. Moreover, the dissolution of intracellular CaOx crystals and the release of intracellular Ca^2+^ and Ox^2−^ are slow, which are completed within a week. Thus, a large amount of Ca^2+^ ions that are toxic to cells will not be generated [68]. That is, cells can use their own defense system to remove CaOx crystals, thereby removing CaOx crystals. Therefore, polysaccharides with better activity can be obtained by sulfation modification to repair damaged cells, improve cell state, and increase the ability of cells to remove CaOx crystals, thereby preventing the retention and deposition of crystals, which can reduce the risk of kidney stone formation [15].

Many reports have shown that polysaccharides can be absorbed into the intestine by oral or intragastric administration, and their effects can reach the kidneys [69,70,71]. Studies have shown that the concentration of polysaccharides in the kidney can reach 60 μg/mL or even higher in animal experiments [72]. Therefore, 60 μg/mL of polysaccharides can be used for in vitro cell research experiments. This study provides strong theoretical data support for subsequent animal experiments.

The formation of urinary calculi induced by hyperoxaluria involves various cell types, including proximal renal tubular epithelial cells, distal renal tubular epithelial cells, and collecting duct cells, in which the ability of UPP to inhibit urinary calculi in other cell types should be further studied. In addition, given its complex structure, the bioactivity, safety, and metabolism of the *U. pinnatifida* polysaccharide must be further investigated in vivo and clinically.

## 5. Conclusions

UPPs can repair oxidatively damaged HK-2 cells. Following UPP repair, cell viability and cell healing ability are enhanced, and the SOD level is increased. In addition, MDA and ROS levels are decreased; the mitochondrial membrane potential is increased; intracellular Ca^2+^ level is decreased; autophagic activity is weakened; lysosomal integrity is improved, and cytoskeleton and cell morphology are improved. In addition, the number of crystals endocytosed by cells through macropinocytosis is greatly increased. UPPs improved the cellular state by repairing damaged subcellular organelles (such as mitochondria, lysosomes, and cytoskeleton) in HK-2 cells and promoted the cellular internalization of nano−COD, which may reduce the risk of calcium oxalate crystal deposition. Compared with the original polysaccharide (UPP0), the sulfated-modified polysaccharides (UPP1, UPP2, and UPP3) had a better repair effect on the cells damaged by oxalate, and UPP2, with a moderate degree of sulfate, had the best effect, which was attributed to the high –OSO_3_^−^ content and low molecular weight of UPP2. Furthermore, UPP2 has shown great application potential in repairing cell damage caused by hyperoxaluria.

## Data Availability

All of the data supporting the results were shown in the study and can be requested from the corresponding author.

## References

[1] Cornell L.D., Amer H., Viehman J.K., Mehta R.A., Lieske J.C., Lorenz E.C., Milliner D.S. (2022). Posttransplant recurrence of calcium oxalate crystals in patients with primary hyperoxaluria: Incidence, risk factors, and effect on renal allograft function. Am. J. Transplant..

[2] Li C.Y., Liu L., Zhao Y.W., Chen J.Y., Sun X.Y., Ouyang J.M. (2021). Inhibition of calcium oxalate formation and antioxidant activity of carboxymethylated *Poria cocos* polysaccharides. Oxid. Med. Cell. Longev..

[3] Schubert G. (2006). Stone analysis. Urol. Res..

[4] Onishi H., Machida Y. (1999). Biodegradation and distribution of water-soluble chitosan in mice. Biomaterials.

[5] Daudon M., Jungers P., Bazin D., Williams J.C. (2018). Recurrence rates of urinary calculi according to stone composition and morphology. Urolithiasis.

[6] Khan S.R., Hackett R.L. (1991). Retention of calcium oxalate crystals in renal tubules. Scanning Microsc..

[7] Khan S.R., Hackett R.L. (1985). Calcium oxalate urolithiasis in the rat: Is it a model for human stone disease? A review of recent literature. Scanning Electron Microsc..

[8] Oliver J., Macdowell M., Whang R., Welt L.G. (1966). The renal lesions of electrolyte imbalance. IV. The intranephronic calculosis of experimental magnesium depletion. J. Exp. Med..

[9] Delatte L.C., Miñón-Cifuentes J., Medina J.A. (1987). New studies on papillary calculi. J. Urol..

[10] Thamilselvan S., Byer K.J., Hackett R.L., Khan S.R. (2000). Free radical scavengers, catalase and superoxide dismutase provide protection from oxalate-associated injury to LLC-PK1 and MDCK cells. J. Urol..

[11] Chanthick C., Thongboonkerd V. (2022). Hyaluronic acid promotes calcium oxalate crystal growth, crystal-cell adhesion, and crystal invasion through extracellular matrix. Toxicol. Vitr..

[12] Kanlaya R., Sintiprungrat K., Chaiyarit S., Thongboonkerd V. (2013). Macropinocytosis is the major mechanism for endocytosis of calcium oxalate crystals into renal tubular cells. Cell Biochem. Biophys..

[13] Chaiyarit S., Singhto N., Thongboonkerd V. (2016). Calcium oxalate monohydrate crystals internalized into renal tubular cells are degraded and dissolved by endolysosomes. Chem. Biol. Interact..

[14] Xiong P., Cheng X.Y., Sun X.Y., Chen XW Ouyang J.M. (2022). Interaction between nanometer calcium oxalate and renal epithelial cells repaired with carboxymethylated polysaccharides. Biomater. Adv..

[15] Huang F., Sun X.Y., Chen X.W., Ouyang J.M. (2021). Effects of selenized astragalus polysaccharide on the adhesion and endocytosis of nanocalcium oxalate dihydrate after the repair of damaged HK-2 cells. ACS Biomater. Sci. Eng..

[16] Yu Y., Zhang Y., Hu C., Zou X., Lin Y., Xia Y., You L. (2019). Chemistry and immunostimulatory activity of a polysaccharide from *Undaria pinnatifida*. Food Chem. Toxicol..

[17] Koh H.S.A., Lu J., Zhou W. (2020). Structural dependence of sulfated polysaccharide for diabetes management: Fucoidan from *Undaria pinnatifida* inhibiting α-glucosidase more strongly than α-amylase and amyloglucosidase. Front. Pharmacol..

[18] Xu X., Zhu X., Lu W., He Y., Wang Y., Liu F. (2019). Effect of sulfated polysaccharide from *Undaria pinnatifida* (SPUP) on proliferation, migration, and apoptosis of human prostatic cancer. Int. J. Polym. Sci..

[19] Xie J.H., Wang Z.J., Shen M.Y., Nie S.P., Gong B., Li H.S., Xie M.Y. (2016). Sulfated modification, characterization and antioxidant activities of polysaccharide from *Cyclocarya paliurus*. Food Hydrocolloid..

[20] Gunasekaran S., Govindan S., Ramani P. (2021). Sulfated modification, characterization and bioactivities of an acidic polysaccharide fraction from an edible mushroom *Pleurotus eous* (Berk.) Sacc. Heliyon.

[21] Ahmad M.M. (2021). Recent trends in chemical modification and antioxidant activities of plants-based polysaccharides: A review. Carbohydr. Polym. Technol. Appl..

[22] Rodrigues J.A.G., Quinderé A.L.G., Benevides N.M.B. (2021). In vitro effects of an *Acanthophora muscoides* (Ceramiales, Rhodophyta) native and modified sulfated polysaccharide fraction on thrombin generation. Acta Sci. Technol..

[23] Yu Y., Song Q., Huang L., Shen M., Yu Q., Chen Y., Xie J. (2020). Immunomodulatory activities of sulfated *Cyclocarya paliurus* polysaccharides with different degrees of substitution on mouse spleen lymphocytes. J. Funct. Foods.

[24] Otero P., Carpena M., Garcia-Oliveira P., Hwang Y., Lee Y.S., Bae S.H., Lee M.S. (2021). Seaweed polysaccharides: Emerging extraction technologies, chemical modifications and bioactive properties. Crit. Rev. Food Sci..

[25] Wang Z., Xie J., Shen M., Nie S., Xie M. (2018). Sulfated modification of polysaccharides: Synthesis, characterization and bioactivities. Trends Food Sci. Tech..

[26] Mukherjee S., Jana S., Khawas S., Kicuntod J., Marschall M., Ray B., Ray S. (2022). Synthesis, molecular features and biological activities of modified plant polysaccharides. Carbohyd. Polym..

[27] Yu Y., Zhu H., Shen M., Yu Q., Chen Y., Xie J. (2021). Sulfation modification enhances the intestinal regulation of *Cyclocarya paliurus* polysaccharides in cyclophosphamide-treated mice via restoring intestinal mucosal barrier function and modulating gut microbiota. Food Funct..

[28] Sun X.Y., Zhang H., Deng J.W., Yu B.X., Zhang Y.H., Ouyang J.M. (2021). Regulatory effects of damaged renal epithelial cells after repair by *Porphyra yezoensis* polysaccharides with different *Sulfation degree* on the calcium oxalate crystal–cell interaction. Int. J. Nanomed..

[29] Chen X.W., Sun X.Y., Tang G.H., Ouyang J.M. (2022). Sulfated *Undaria pinnatifida* polysaccharide inhibits the formation of kidney stones by inhibiting HK-2 cell damage and reducing the adhesion of nano-calcium oxalate crystals. Biomater. Adv..

[30] Sun X.Y., Ouyang J.M., Liu A.J., Ding Y.M., Li Y.B., Gan Q.Z. (2015). Preparation, characterization, and in vitro cytotoxicity of COM and COD crystals with various sizes. Mater. Sci. Eng. C.

[31] Ladrech S., Eybalin M., Puel J.L., Lenoir M. (2017). Epithelial-mesenchymal transition, and collective and individual cell migration regulate epithelial changes in the amikacin-damaged organ of Corti. Histochem. Cell. Bio..

[32] Lu M., Wang J., Ren G., Qin F., Zhao Z., Li K., Lin Y. (2022). Superoxide-like Cu/GO single-atom catalysts nanozyme with high specificity and activity for removing superoxide free radicals. Nano Res..

[33] Buwono N.R., Risjani Y., Soegianto A. (2022). Oxidative stress responses of microplastic-contaminated gambusia affinis obtained from the Brantas River in East Java, Indonesia. Chemosphere.

[34] Rao C.Y., Sun X.Y., Ouyang J.M. (2019). Effects of physical properties of nano-sized hydroxyapatite crystals on cellular toxicity in renal epithelial cells. Mater. Sci. Eng. C.

[35] Guan B., Jiang Y.T., Lin D.L., Lin W.H., Xue H.W. (2022). Phosphatidic acid suppresses autophagy through petitive inhibition by binding GAPC (glyceraldehyde-3-phosphate dehydrogenase) and PGK (phosphoglycerate kinase) proteins. Autophagy.

[36] Sudhakar J.N., Lu H.H., Chiang H.Y., Suen C.S., Hwang M.J., Wu S.Y., Shui J.W. (2020). Lumenal Galectin-9-Lamp2 interaction regulates lysosome and autophagy to prevent pathogenesis in the intestine and pancreas. Nat. Commun..

[37] Chen X.W., Huang W.B., Sun X.Y., Xiong P., Ouyang J.M. (2021). Antioxidant activity of sulfated *Porphyra yezoensis* polysaccharides and their regulating effect on calcium oxalate crystal growth. Mater. Sci. Eng. C.

[38] Sun X.Y., Gan Q.Z., Ouyang J.M. (2017). Size-dependent cellular uptake mechanism and cytotoxicity toward calcium oxalate on Vero cells. Sci. Rep..

[39] Mas-Bargues C., García-Domínguez E., Borrás C. (2022). Recent approaches to determine static and dynamic redox state-related parameters. Antioxidants.

[40] Zhao M., Wang Y., Li L., Liu S., Wang C., Yuan Y., Liu J. (2021). Mitochondrial ROS promote mitochondrial dysfunction and inflammation in ischemic acute kidney injury by disrupting TFAM-mediated mtDNA maintenance. Theranostics.

[41] Joshi S., Khan S.R. (2019). Opportunities for future therapeutic interventions for hyperoxaluria: Targeting oxidative stress. Expert Opin. Ther. Targets..

[42] Khan S.R. (2013). Reactive oxygen species as the molecular modulators of calcium oxalate kidney stone formation: Evidence from clinical and experimental investigations. J. Urol..

[43] Han Y., Zhao M., Ouyang K., Chen S., Zhang Y., Liu X., Wang W. (2021). Sulfated modification, structures, antioxidant activities and mechanism of *Cyclocarya paliurus* polysaccharides protecting dendritic cells against oxidant stress. Ind. Crop. Prod..

[44] Zhao B., Tao F., Wang J., Zhang J. (2020). The sulfated modification and antioxidative activity of polysaccharides from *Potentilla anserine* L.. New J. Chem..

[45] Li Z., Wei Y., Wang Y., Zhang R., Zhang C., Wang C., Yan X. (2022). Preparation of highly substituted sulfated alfalfa polysaccharides and evaluation of their biological activity. Foods.

[46] Chen Y., Zhang H., Wang Y., Nie S., Li C., Xie M. (2015). Sulfated modification of the polysaccharides from ganoderma atrum and their antioxidant and immunomodulating activities. Food Chem..

[47] Wang J., Hu S., Nie S., Yu Q., Xie M. (2016). Reviews on mechanisms of in vitro antioxidant activity of polysaccharides. Oxidative Med. Cell. Longev..

[48] Marrache S., Dhar S. (2015). The energy blocker inside the power house: Mitochondria targeted delivery of 3-bromopyruvate. Chem. Sci..

[49] Kuznetsov A.V., Javadov S., Margreiter R., Grimm M., Hagenbuchner J., Ausserlechner M.J. (2019). The role of mitochondria in the mechanisms of cardiac ischemia-reperfusion injury. Antioxidants.

[50] Nakamura S., Shigeyama S., Minami S., Shima T., Akayama S., Matsuda T., Yoshimori T. (2020). LC3 lipidation is essential for TFEB activation during the lysosomal damage response to kidney injury. Nat. Cell Biol..

[51] Mulay S.R., Honarpisheh M.M., Foresto-Neto O., Shi C., Desai J., Zhao Z.B., Anders H.J. (2019). Mitochondria permeability transition versus necroptosis in oxalate-induced AKI. J. Am. Soc. Nephrol..

[52] Park K., Lim H., Kim J., Hwang Y., Lee Y.S., Bae S.H., Lee M.S. (2022). Lysosomal Ca^2+^-mediated TFEB activation modulates mitophagy and functional adaptation of pancreatic β-cells to metabolic stress. Nat. Commun..

[53] Lumlertgul N., Siribamrungwong M., Jaber B.L., Jaber B.L., Susantitaphong P. (2018). Secondary oxalate nephropathy: A systematic review. Kidney Int. Rep..

[54] Sun X.Y., Zhang H., Liu J., Ouyang J.M. (2019). Repair activity and crystal adhesion inhibition of polysaccharides with different molecular weights from red algae *Porphyra yezoensis* against oxalate-induced oxidative damage in renal epithelial cells. Food Funct..

[55] Jang S., Javadov S. (2017). Association between ROS production, swelling and the respirasome integrity in cardiac mitochondria. Arch. Biochem. Biophys..

[56] Shen X., Tang Z., Bai Y., Wan M., Yu M., Chen J., Ge M. (2022). Astragalus polysaccharide protects against cadmium-induced autophagy injury through reactive oxygen species (ROS) pathway in chicken embryo fibroblast. Biol. Trace Elem. Res..

[57] Huang F., Chen J.Y., Ouyang J.M. (2020). Comparison of the inhibition of high phosphate-induced smooth muscle cell calcification by porphyra yezoensis and astragalus polysaccharides. J. Funct. Foods.

[58] Li M., Luo T., Huang Y., Su J., Li D., Chen X., Zhang Y., Huang L., Li S., Jiao C. (2020). Polysaccharide from pycnoporus sanguineus ameliorates dextran sulfate sodium-induced colitis via helper T cells repertoire modulation and autophagy suppression. Phytother. Res..

[59] Li C., Duan S., Li Y., Pan X., Han L. (2021). Polysaccharides in natural products that repair the damage to intestinal mucosa caused by cyclophosphamide and their mechanisms: A review. Carbohyd. Polym..

[60] Ying L., Pan Y., Wang Y., Xu P. (2017). Physicochemical properties, in vitro antioxidant activities and protective effects of *Liubao tea* polysaccharides on HUVEC. J. Tea Sci..

[61] Dinoro J., Maher M., Talebian S., Jafarkhani M., Mehrali M., Orive G., Dolatshahi-Pirouz A. (2019). Sulfated polysaccharide-based scaffolds for orthopaedic tissue engineering. Biomaterials.

[62] Wu D.T., He Y., Fu M.X., Gan R.Y., Hu Y.C., Peng L.X., Zou L. (2022). Structural characteristics and biological activities of a pectic-polysaccharide from okra affected by ultrasound assisted metal-free fenton reaction. Food Hydrocolloid..

[63] Zhang L., Ma L., Pan Y., Zheng X., Sun Q., Wang Z., Qiao H. (2021). Effect of molecular weight on the antibacterial activity of polysaccharides produced by *Chaetomium globosum* CGMCC 6882. Int. J. Biol. Macromol..

[64] Saravana P.S., Cho Y.N., Patil M.P., Cho Y.J., Kim G.D., Park Y.B., Chun B.S. (2018). Hydrothermal degradation of seaweed polysaccharide: Characterization and biological activities. Food Chem..

[65] Tao Y., Ma J., Huang C., Lai C., Ling Z., Yong Q. (2022). The immunomodulatory activity of degradation products of *Sesbania cannabina* galactomannan with different molecular weights. Int. J. Biol. Macromol..

[66] Han J., Guo D., Sun X.Y., Wang J.M., Ouyang J.M., Gui B.S. (2019). Repair effects of astragalus polysaccharides with different molecular weights on oxidatively damaged HK-2 cells. Sci. Rep..

[67] Zuo T., Li X., Chang Y., Duan G., Yu L., Zheng R., Tang Q. (2015). Dietary fucoidan of acaudina molpadioides and its enzymatically degraded fragments could prevent intestinal mucositis induced by chemotherapy in mice. Food Funct..

[68] Lieske J.C., Norris R., Swift H., Toback F.G. (1997). Adhesion, internalization and metabolism of calcium oxalate monohydrate crystals by renal epithelial cells. Kidney Int..

[69] Yu H.D., Chun C., Xiong F., Rui H.L. (2021). Study on the pharmacokinetics of mulberry fruit polysaccharides through fluorescence labeling. Int. J. Boil. Macromol..

[70] Li F., Wei Y., Zhao J., Zhang L., Li Q. (2022). In vivo pharmacokinetic study of a *Cucurbita moschata* polysaccharide after oral administration. Int. J. Boil. Macromol..

[71] Xia H., Yang C., Zhou B., Tang H., Yang L., Liao W., Sun G. (2021). Pharmacokinetics and Excretion Study of *Lycium barbarum* Polysaccharides in Rats by FITC-Fluorescence Labeling. Foods.

[72] Lin X., Wang Z., Sun G., Shen L., Xu D., Feng Y. (2010). A sensitive and specific HPGPC-FD method for the study of pharmacokinetics and tissue distribution of Radix Ophiopogonis polysaccharide in rats. Biomed. Chromatogr..

